# Measurement of caveolin-1 densities in the cell membrane for quantification of caveolar deformation after exposure to hypotonic membrane tension

**DOI:** 10.1038/s41598-017-08259-5

**Published:** 2017-08-10

**Authors:** Masashi Tachikawa, Nobuhiro Morone, Yosuke Senju, Tadao Sugiura, Kyoko Hanawa-Suetsugu, Atsushi Mochizuki, Shiro Suetsugu

**Affiliations:** 1 Theoretical Biology Laboratory, RIKEN, 2-1 Hirosawa, Wako, 351-0198 Japan; 2 MRC Toxicology Unit, University of Leicester, Leicester LE1 9HN, UK, and Institute for Integrated Cell-Material Sciences (iCeMS), Kyoto University, Kyoto, 606-8501 Japan; 3 Graduate School of Information Science, Nara Institute of Science and Technology, Ikoma, 630-0192 Japan; 4 Institute of Biotechnology, University of Helsinki, Helsinki, 00014 Finland; 5 Graduate School of Biological Sciences, Nara Institute of Science and Technology, Ikoma, 630-0192 Japan; 6 Core Research for Evolutionary Science and Technology, Japan Science and Technology Agency, Kawaguchi, 332-0012 Japan

## Abstract

Caveolae are abundant flask-shaped invaginations of plasma membranes that buffer membrane tension through their deformation. Few quantitative studies on the deformation of caveolae have been reported. Each caveola contains approximately 150 caveolin-1 proteins. In this study, we estimated the extent of caveolar deformation by measuring the density of caveolin-1 projected onto a two-dimensional (2D) plane. The caveolin-1 in a flattened caveola is assumed to have approximately one-quarter of the density of the caveolin-1 in a flask-shaped caveola. The proportion of one-quarter-density caveolin-1 increased after increasing the tension of the plasma membrane through hypo-osmotic treatment. The one-quarter-density caveolin-1 was soluble in detergent and formed a continuous population with the caveolin-1 in the caveolae of cells under isotonic culture. The distinct, dispersed lower-density caveolin-1 was soluble in detergent and increased after the application of tension, suggesting that the hypo-osmotic tension induced the dispersion of caveolin-1 from the caveolae, possibly through flattened caveolar intermediates.

## Introduction

The plasma membrane, composed of amphipathic lipid molecules, exhibits two-dimensional fluidic properties that allow for flexible responses against tension without breakage. The mechanisms involved in the plasma membrane response to tension are not fully understood. The increase in cell surface area is mediated, at least in part, by the disassembly of membrane reservoirs, which are areas of folded membrane that can be flattened^1–5^. Caveolae function as membrane reservoirs that can be flattened after an increase in membrane tension. In the resting state, caveolae are stable flask- or cup-shaped plasma membrane invaginations with diameters of approximately 100 nm^6, 7^.

**Figure 1:**
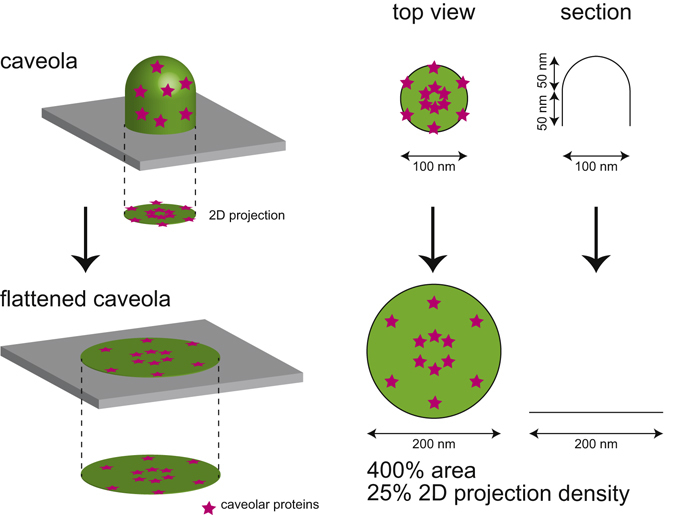
The expected density of caveolin-1 in a two-dimensional projection of a flattened caveola. A caveola typically has a 100 nm diameter and a 100 nm depth. We approximated a caveola as a cylinder with a 50 nm depth and a 100 nm diameter, capped with a 100 nm diameter hemisphere. The entire area of this structure is unfolded into a flat membrane and the two-dimensional, projected area is calculated to be increased 4-fold. Therefore, the density of molecules associated with the two-dimensionally projected caveola, such as caveolin-1, is expected to be decreased to 25% of the density of molecules associated with the folded caveola.

There are approximately 150 caveolin-1 molecules associated with the flask- or cup-shaped caveola membrane^8, 9^. A typical caveola has an approximate depth of 100 nm and diameter of 100 nm; thus, a caveola can be represented as a cylinder of 50 nm length with a hemispheric cap radius of 50 nm (Fig. 1). If we set the radius as *r* and the length of the cylinder as *r*, then the calculated surface area of the cylinder is 2*πr* × *r* + 0.5 × 4*πr* ^2^ = 4*πr* ^2^. If this cylinder with its cap is flattened by tension, then the diameter projected onto the plane would increase 4-fold because the area of the original projection is *πr* ^2^. Therefore, although the density of caveolin-1 on the membrane is not expected to change during caveolar flattening, the projection density, which is the density of caveolin-1 projected onto the plane parallel to the plasma membrane, is assumed to decrease. Here, a flattened caveola has a 200 nm diameter (Fig. 1), and therefore the appearance of one-quarter-density caveolin-1 is expected after the application of tension if caveolin-1 remains in the caveola.

In this study, we labelled caveolin-1 using antibody staining or Dendra2, a photoswitchable fluorescent protein^10^. The antibody staining requires the solubilization of the membrane by detergent, i.e., partial disruption of the membrane, to allow the antibody access to caveolin-1. By contrast, Dendra2 can be visualized without solubilization of the membrane. We determined the precise localization of caveolin-1 by stochastic localization methods: stochastic optical reconstruction microscopy (STORM) for antibody labelling and photoactivated localization microscopy (PALM) for Dendra2. Both methods determined the coordinates of caveolin-1 localization. We then evaluated the projection density of caveolin-1 using neighbour distance measurements. Using these densities, we quantitatively analysed the populations of caveolin-1 in density ranges corresponding to the shapes of the caveolae. When using antibody labelling, we did not detect any increase in the population of caveolin-1 corresponding to flattened caveolae after hypo-osmotic treatment, which are expected to have one-quarter the projection density of typical caveolae under isotonic conditions. However, we detected an increase in the population of the one-quarter-density caveolin-1 using caveolin-1-Dendra2. The one-quarter-density caveolin-1 was not a distinct population from the typical density caveolin-1. In both the observations using antibody and the observations using Dendra2, we detected an increase in the dispersed caveolin-1 population with lower density than the population with one-quarter density. Therefore, hypo-osmotic tension induced caveolar deformation and dispersion, and the deformed caveolae contained caveolin-1 that is more soluble in detergent.

## Results

### Observation of caveolin-1 by super resolution microscopy using antibody labelling

**Figure 2:**
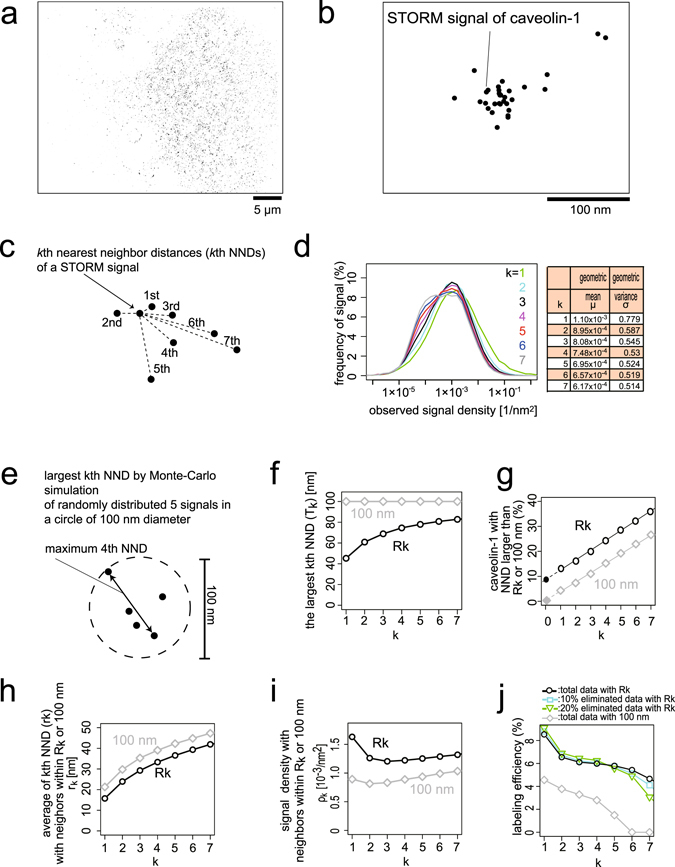
The *k* ^th^ nearest neighbouring distance (NND) measurement using a typical caveolin-1 observation from a cell. (**a**,**b**) Representative STORM images of a whole cell (**a**) and a caveola (**b**) with caveolin-1 staining obtained by indirect immunofluorescence with an anti-caveolin-1 antibody. Each dot corresponds to one STORM signal. (**c**) Schematic diagram of the *k* ^th^ NND for the signal density estimation of caveolin-1. (**d**) The representative density distributions of the STORM signals calculated from the *k* ^th^ NND in a cell under isotonic conditions, for *k* = 1–7 on a logarithmic scale. In the table, the geometric means (*μ*) and geometric variances (*σ*) are shown after fitting with a single log-normal distribution, because of their unimodal distribution. (**e**) A schematic diagram showing the maximum calculations of the 4^th^ NND using the 5 (4 + 1) signals generated by a Monte-Carlo simulation in a 100 nm diameter circle, determined from the typical diameter of a caveola. (**f**) The maximum distances for the *k* ^th^ NND values (T_k_ [nm]) for given *k* values, estimated with Monte-Carlo simulations (*R*_*k*_) as in (**e**), are shown in black. Without the simulation, the maximum value was hypothesized to be the caveolar diameter of 100 nm and is shown in grey. (**g**) A plot of STORM signal percentages that did not have *k* signals within the range *T*_*k*_ for each *k* value. Calculations with maximums, which were a caveolar diameter of 100 nm and the maximum estimated by Monte-Carlo simulations (*R*_*k*_), are shown in grey and black, respectively. The plots of percentages on *k* were approximated well within linear equations (grey and black lines). The intercepts of these lines (*k* = 0) were assumed to be the percentages of caveolin-1 outside the caveolin-1 assembly regions and were −2.45% (grey-filled diamond) and 8.53% (black-filled circle), with the assumption of uniform observation efficiency. (**h**) Averages of *k* ^th^ NND (r_k_ [nm]) after the elimination of signals with *R*_*k*_ or 100 nm maximum values, shown in black and grey, respectively. (**i**) Estimated signal densities inside caveolae, calculated using equation (1) and the averages of the *k* ^th^ NND within caveolae in (**h**). (**j**) Observation efficiency estimations of caveolin-1 with the *k* ^th^ NND plotted against *k*, after the elimination of signals as in (**e**,**f**,**g**), with *R*_*k*_ or 100 nm maximum distances. To examine the robustness of the estimation, the observation efficiencies calculated from the data, in which 10 or 20% of the signals were further randomly eliminated from the original data, were also calculated after dividing by 0.9 or 0.8 for normalization to the observation efficiency from the original data. The increase in *k* exceeds the assumed number of signals per caveola, and thus the observation efficiency is underestimated.

First, we observed individual endogenous caveolin-1 molecules by antibody-based STORM observation (Fig. 2a,b). After fixation of the cells, the plasma membrane was solubilized using detergent to allow the antibody to access the caveolin-1 proteins. Then, we labelled endogenous caveolin-1 with an anti-caveolin-1 antibody followed by a secondary antibody double-labelled with Alexa 405 and Alexa 647, to obtain the laser-induced activation of the Alexa 647 dye necessary for single-molecule resolution^11, 12^. Because the Alexa 647 dye emits multiple signals, we recorded the first frame by using a 405 nm laser for activation, followed by three frames using a 633 nm laser for observation. Only the signals that appeared in both the 1^st^ and 2^nd^ frames were considered to represent the activation-induced signals from the antibodies.

We first evaluated the STORM signals resulting from caveolin-1 staining. The STORM data obtained without the anti-caveolin-1 antibody or from the cells treated with siRNA for caveolin-1 had almost no STORM signal (Figure S1), thus suggesting that our observations by STORM represented the distribution of caveolin-1. The accuracy of the STORM signal in the x-y dimension is within the range of approximately 20 nm, whereas that of the z dimension is in the range of approximately 50 nm^12^. Thus, the depth of each caveola, which is approximately 100 nm, could not be observed by STORM. Therefore, we analysed the x and y coordinates to calculate the two-dimensional projection density (Fig. 2).

We calculated the projection density of caveolin-1 signals and attempted to identify the populations of free, disassembled caveolin-1 outside caveolae and the population of the assembled caveolin-1 within caveolae. We converted the coordinates of the caveolin-1 signals by STORM to the projection density of each caveolin-1 molecule. There were two challenges to estimating the actual projection density of caveolin-1 from the observed STORM coordinates. First, the calculation of the observed density depended on the setting of the area used to calculate the density. Second, the observed signals represented only a portion of the caveolin-1 molecules, and the observation efficiency of the caveolin-1 molecules, i.e., the number of observed caveolin-1 signals as a ratio of the total caveolin-1 molecules, was not known.

For coordinate distribution analysis, Ripley’s K function is widely used to examine the randomness in signal distribution^13^; however, it is difficult to convert to signal densities. Cluster analysis and location-adaptive density estimation have also been used to examine signal distribution^14, 15^. However, we found that these two methods were ineffective when two caveolae were close to each other. Therefore, we developed a method to estimate the labelling efficiency and density of caveolin-1 based on the *k* ^th^ nearest neighbour distance (*k* ^th^ NND). From a given caveolin-1 signal, we measured the distance to its neighbouring caveolin-1 signals (Fig. 2c)^16^; we then converted the distances to the projection densities.

### The *k*^th^ NND analysis for density measurement

The two-dimensional distance, in this case, *k* ^th^ NND, reflected the projection density of the signals. We selected a cell in isotonic culture medium, Dulbecco’s Modified Eagle’s medium (DMEM) supplemented with 10% serum, to evaluate the NND measurement for density estimation. We selected an area of 8,262 nm × 7,160 nm, in which the signal density was high and caveolae were thought to be densely distributed. Assuming that the local signal distribution was random and approximated by a Poisson point process, we estimated the local projection density (${\rho }_{k}^{i}$) around the signal *i* from its *k* ^th^ NND ${r}_{k}^{i}$,

1 $${\rho }_{k}^{i}=\frac{1}{\pi {r}_{k}^{{i}^{2}}}{\{\frac{{\rm{\Gamma }}(k+1)}{{\rm{\Gamma }}(k+\frac{1}{2})}\}}^{2}$$

where Γ(·) is the gamma function^16^. We then calculated the 1^st^, 2^nd^, 3^rd^, 4^th^, 5^th^, 6^th^, and 7^th^ (*k* = 1–7) NNDs for all observed signals. The frequency distributions of ${\rho }_{k}^{i}$ (equation (1)) for *k* = 1–7 were plotted on a logarithmic scale with the geometric means (*μ*) and geometric variances (*σ*) (Fig. 2d). The similar distributions of the *k* ^th^ NND from *k* = 1–7 implied that equation (1) provided a robust measurement of the local signal densities.

### Estimation of observation efficiency and optimal *k* value for *k*^th^ NND

In general, the estimation of the molecule density by the *k* ^th^ NND with a smaller *k* is influenced strongly by spatial randomness and shows large fluctuations. Thus, the larger *k* is thought to result in accurate estimation of the density. However, when we considered the density of the molecules in specific subcellular organelles, then the distance between the molecules has the maximum distance that is determined by the shape of the subcellular organelles and by the observation efficiency. If the observation efficiency is low, then the average number of signals in the subcellular structure is small, and a *k* ^th^ NND with a large *k* would have resulted in the measurement of the distance between signals in two distant subcellular structures, which would not reflect the signal density for the subcellular structure. Having the maximum distance shorter than the *k* ^th^ NND could occur, especially if a large *k* value is used. Therefore, we estimated the observation efficiency and the optimal *k* value.

First, we considered a typical caveola as having a 100 nm diameter and 150 membrane-associated caveolin-1 molecules. The projection density of caveolin-1 in this assembly was 0.019 [1/nm^2^]. Second, we considered the maximum distance, which is the possible largest *k* ^th^ NND within a caveola, to avoid the usage of signals outside of the caveola for *k* ^th^ NND estimation. The maximum distance (*T*_*k*_) for the *k* ^th^ NND data are defined as the largest distance between two signals within one caveola that has *k* + *1* signals because more than *k* signals are necessary to measure the *k* ^th^ NND within one caveola, and because an increase in the number of signals in a limited area (a caveola) decreases the expected value of the largest *k* ^th^ NND. There were two candidates for the maximum distance values. One candidate was the hypothesized caveolar diameter, *T*_*k*_ = 100 nm, which is shown in grey in Fig. 2f–j. Another candidate was a typical value of the largest distance estimated by a Monte-Carlo simulation (*T*_*k*_ = *R*_*k*_), which is shown in black in Fig. 2f–j. In a Monte-Carlo trial, we randomly generated *k* + *1* dots (signals) inside a circle with a diameter of 100 nm and took the largest *k* ^th^ NND (Fig. 2e). We repeated the trial 10,000 times and took the average of the largest *k* ^th^ NND as a typical value of the largest distance, *R*_*k*_ (Fig. 2f). The estimations of the maximum values are shown in Fig. 2f, and the percentages of eliminated *k* ^th^ NND data with both maximum values are shown in Fig. 2g.

After the elimination of the *k* ^th^ NND larger than the maximum distance, the average of the *k* ^th^ NND (${\bar{r}}_{k}$) (Fig. 2h) was substituted into equation (1), and the projection density (*ρ*_*k*_) of signals within a caveola was obtained. The signal density within a caveola was calculated from the observation efficiency *p*_*k*_ by using the following equation:

2 $${\rho }_{k}=\frac{1}{\pi \cdot {50}^{2}}\sum _{n=k+1}^{150}n\cdot \frac{{(150{p}_{k})}^{n}{e}^{-150{p}_{k}}}{\alpha \cdot n!}$$

where $\alpha =\sum _{m=k+1}^{150}\frac{{(150{p}_{k})}^{m}{e}^{-150{p}_{k}}}{m!}$ is the normalization factor. Here, we assumed that the number of labelled caveolin-1 molecules was represented by a Poisson distribution with λ = 150*p*_*k*_ with a caveolar radius of 50 nm. Solving this equation numerically, we obtained an observation efficiency *p*_*k*_ for the two kinds of maximum distances (*T*_*k*_) (Fig. 2j). The maximum distance determined by the Monte-Carlo simulation (*T*_*k*_ = *R*_*k*_) gave a stable estimation of approximately 6% for broader *k* values (*k* = 2–6). In contrast, the maximum distance of 100 nm (*T*_*k*_ = 100 nm) did not provide a robust estimation of the observation efficiency (Fig. 2j). Because the *k* ^th^ NND with different *k* values reflects the signal density independently of *k* values under *R*_*k*_, the estimation of NND by *R*_*k*_ is a more robust estimation than that using a maximum distance of 100 nm.

We then assessed the difference between the two maximum distances, 100 nm and *R*_*k*_ using a Monte-Carlo simulation. If our assumptions that the typical distance between caveolae was more than 100 nm and that there are few caveolin-1 molecules outside of caveolae were correct, then the maximum distances by both 100 nm and the Monte-Carlo simulation would eliminate the *k* ^th^ NND data that did not belong to caveolae, where both estimations of the observation efficiency would be accurate. Apparently, the maximum distance simply set to 100 nm did not give a more robust estimation of labelling than that of the Monte-Carlo simulation. Thus, either one or both of the two assumptions were incorrect. In fact, several electron micrographs revealed caveola-rich plasma membrane compartments within which the distances between caveolae were less than 100 nm, thus challenging the first assumption. For the second assumption, the caveolin-1 signal at the plasma membrane outside of caveolae was considered to be a background signal using conventional microscopy analyses. However, the second assumption was apparently incorrect based on STORM analyses. Moreover, our method estimated the percentage of caveolin-1 outside of caveolae by the intercept (k = 0) of the linear fitting of eliminated signals (Fig. 2g). The estimated percentage of caveolin-1 outside of caveolae with *T*_*k*_ = 100 nm gave a negative value (−2.45%), which is impossible and thus suggested the inaccuracy of this estimation. In contrast, the estimation with *T*_*k*_ = *R*_*k*_ provided a reasonable value of 8.53%, consistent with fractionation data showing a small fraction of caveolin-1 outside of the caveolar coat protein complex^9^. Because the maximum value calculated by the Monte-Carlo method was less than 100 nm, the maximum value eliminated many *k* ^th^ NND data, and we assumed that the remaining data after elimination were more reliable for the density estimation.

To further confirm the validity of this method, we estimated the dependency of the *k* ^th^ neighbouring method on the spatial pattern of the signal distribution. We randomly eliminated 10 or 20% of the signals to evaluate the fluctuation of the labelling by the antibody and performed the same calculations. The calculated labelling efficiencies were divided by 0.9 or 0.8 to compensate for the decrease in the signals and plotted in Fig. 2j. The estimation of the labelling efficiencies was not significantly different across *k* = 1–5, thereby suggesting the validity of this method. Thus, we assumed that *k* = 4 or 5 would be optimal for the estimation of the caveolin-1 projection density.

The estimation of the observation efficiency of caveolin-1 was ~6% for this cell (*k* = 4). Approximately 9 caveolin-1 antibody molecules per typical caveola (150 caveolin-1 molecules) were estimated to be observed by STORM, which is equivalent to a projection density of 1.1 · 10^−3^ [1/nm^2^]. This estimation was consistent with previously published data demonstrating that each caveola was labelled with 5–10 caveolin-1 antibodies, as determined by an immuno-electron-microscopy after freeze-fracture procedures^17^. With the assumption of a Poisson distribution, 95.7% of caveolae were estimated to be observed with 4–15 antibody molecules. Interestingly, the mode density of the frequency distribution of the signals under isotonic conditions varied by 1.2 ± 0.33 · 10^−3^ [1/nm^2^] among 8 cells (*k* = 4), a result supporting similar labelling of caveolin-1 between experiments. However, the variation in the mode-density values (0.3/1.12 · 100 ≃ 28%) is notably large for the detection of the density-dependent responses of caveolin-1 distribution to hypotonic treatment, which is expected to decrease the density to 25%.

### The density of caveolin-1 in cells under hypo-osmolarity conditions

To examine the changes in caveolin-1 density during hypotonic treatment, we analysed the signal densities of caveolin-1 from sets of 6 cells placed in hypotonic medium for either 3 or 5 min in comparison with the aforementioned 8 cells that were placed in isotonic medium. Assuming the mode of caveolin-1 density in each similarly treated cell is the same, i.e., the caveolin-1 density in typical caveolae, we calculated the geometric mean of the mode densities of the signals among cells under the same treatment (${\bar{{\varrho }}}_{{\rm{treat}}}$, where treat = iso, 3 min, 5 min). Then, we adjusted the density scale of each cell by multiplying the ratio of the geometric mean of the mode densities of the signals and the mode density of each cell (${\bar{{\varrho }}}_{{\rm{treat}}}/{{\varrho }}_{{\rm{treat}}}^{i}$, where *i* is the index of each cell). This method compensated for the experimental fluctuation among the cells we observed. These adjusted density distributions precisely described the relative density distributions of caveolin-1. This made it possible to detect the change in the distribution, which was otherwise concealed by the experimental variation, i.e., 28% variation in the observed density.

**Figure 3:**
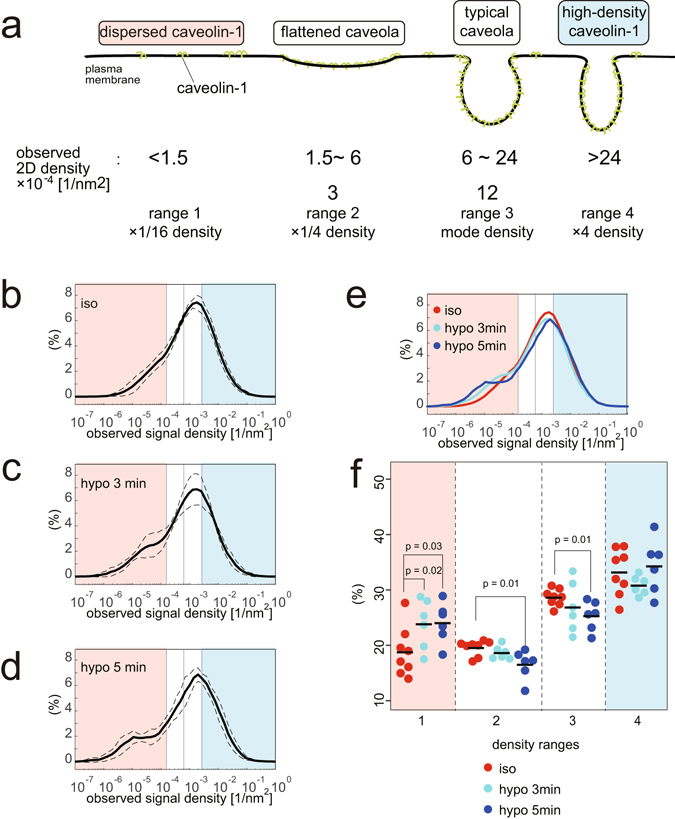
Local density estimation from STORM signals of caveolin-1. (**a**) A schematic representation of caveolin-1 density and expected shapes of caveolae by a 4-times density scale. Under isotonic conditions, typical caveolae had a mode density of 12 × 10^−4^/nm^2^ and flattened caveolae were expected to have a density of 3 × 10^−4^/nm^2^. Therefore, we analysed the percentages of caveolin-1 molecules by densities ranging from 1.5 to 6 and from 6 to 24 × 10^−4^/nm^2^ as “flattened” and “typical” caveolae, respectively. The percentages of caveolin-1 below 1.5 (dispersed caveolin-1) and above 24 (high-density caveolin) were also analysed. (**b**–**e**) The average density distributions of the STORM signals by the 4^th^ NND in cells treated with (**b**) isotonic culture medium, (**c**) hypotonic conditions for 3 min and (**d**) hypotonic conditions for 5 min. The distribution of each cell was analysed using relative densities to the mode density and then plotted using the averaged mode. The dashed line indicates the standard deviation. The density ranges in (**a**) are also indicated by colour. In (**e**), the superposition of (**b**–**d**) is shown. (**f**) Percentages of caveolin-1 by the density ranges as in (**a**) in each cell. The average percentages (black bars) between cells in isotonic medium and hypotonic treatments for 3 and 5 min were significantly different. Indicated p-values were obtained using the Student’s t-test.

Our objective was to detect the behaviours of the caveolin-1 population with one-quarter-density compared with the mode density, which was considered the typical caveolar density. Therefore, we configured four ranges of projection densities: the first range $(\rho \le {\bar{{\varrho }}}_{{\rm{iso}}}/8)$, second range $({\bar{{\varrho }}}_{{\rm{iso}}}/8 < \rho \le {\bar{{\varrho }}}_{{\rm{iso}}}/2)$, third range $({\bar{{\varrho }}}_{{\rm{iso}}}/2 < \rho \le 2{\bar{{\varrho }}}_{{\rm{iso}}})$, and fourth range $(2{\bar{{\varrho }}}_{{\rm{iso}}} < \rho )$. The representative densities of the second and the third range are ${\bar{{\varrho }}}_{{\rm{iso}}}/4\simeq 3[{10}^{-4}{/\mathrm{nm}}^{2}]$ and ${\bar{{\varrho }}}_{{\rm{iso}}}\simeq 12[{10}^{-4}{/\mathrm{nm}}^{2}]$, respectively (Fig. 3a). Therefore, the first through to the fourth range were designed to detect the caveolin-1 scattered over the plasma membrane (dispersed caveolin-1), the caveolin-1 at one-quarter-density (flattened caveolar density), the caveolin-1 at the mode density (the typical caveolar density), and the caveolin-1 at a higher density, respectively. The signals were then apportioned to the four ranges based on their surrounding signal densities calculated with the 4^th^ NND and the percentages of signals in these ranges were calculated.

The averaged density distributions of caveolin-1 detected by antibody labelling are shown in Fig. 3b–e. After hypotonic treatment, the percentages of caveolin-1 at the typical caveolar density (in the third range) decreased and, surprisingly, the percentage of caveolin-1 at the flattened caveolar density (in the second range) did not increase but decreased. Interestingly, the percentage of dispersed caveolin-1 (in the first range) significantly increased (Fig. 3f). These changes were more evident with the 5-min hypo-osmotic treatment than with the 3-min hypo-osmotic treatment. The percentage of the high-density caveolin-1 (in the fourth range) was not significantly changed.

### Ripley’s L function analysis to examine the correlation between caveolin-1 populations

**Figure 4:**
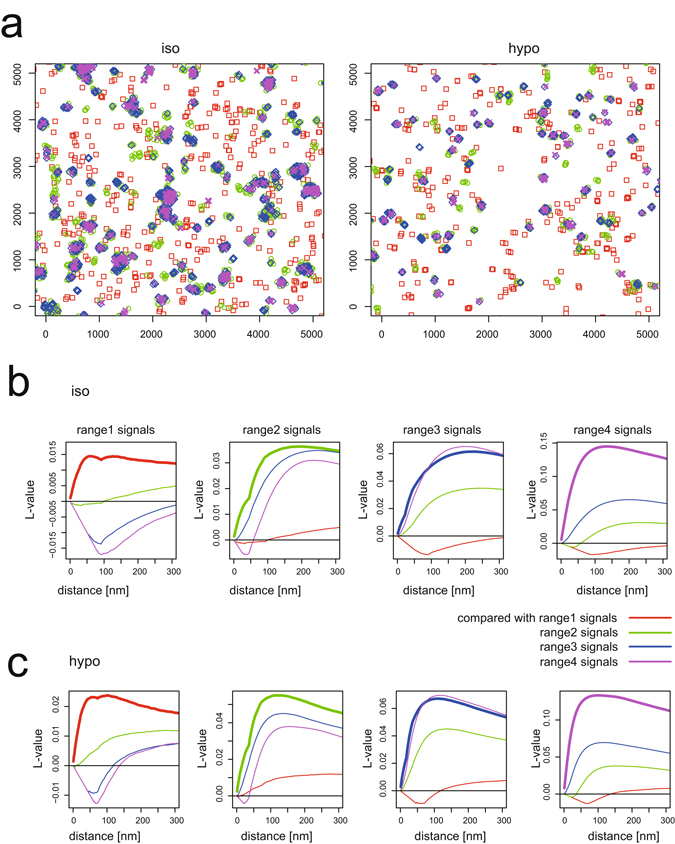
Ripley L function analysis to examine correlation between caveolin-1 populations, showing district dispersed caveolin-1 population from the others. (**a**) Typical representation of STORM signals from cells under isotonic and hypo-osmotic conditions for 5 min in Fig. 3. Density ranges are distinguished by colour: red, green, blue and magenta dots represent signals in the first, second, third and fourth range, respectively. (**b**,**c**) Multivariate Ripley’s L function analysis to examine the correlation between caveolin-1 from cells under (**b**) isotonic and (**c**) hypo-osmotic conditions in the four ranges in Fig. 3a. The leftmost panels show L function analysis with signals in the first range; a bold red line indicates autocorrelation among signals in the first range, and thin lines show cross-correlations among signals in the first range and in the second (green), third (blue) and fourth (magenta) ranges. The second, third and fourth panel shows L function analysis with signals in the second, third and fourth range, respectively. The analysis of the other cells in Fig. 3 is on Figure S1.

Next, we evaluated the localization of caveolin-1 associated with the four ranges. We plotted the caveolin-1 coordinates coloured by range (Fig. 4a). The spatial relationships between caveolin-1 signals in the four ranges were then analysed by the multivariate Ripley’s L function^18^ (Figs 4b,c and S1). These graphs represent spatial correlation between the signals from the *i*th and *j*th range (*i* and *j* are 1–4). First, autocorrelations (bold lines) are positive and larger than any other cross-correlations (thin lines) for the signals from all four ranges, indicating that the observed area can be decomposed into patches in which signals from one of the four ranges are dominant. In particular, the localization signals of the first range were mutually exclusive with the signals in other ranges (cross-correlations are almost zero or negative) (Fig. 4a,b,c). By contrast, the caveolin-1 in the three ranges (the second, third and fourth ranges) localized close to each other (cross-correlations are positive), which indicated that the caveolin-1 in these densities formed a continuous structure in cells under both isotonic conditions and hypotonic treatment (Fig. 4b,c). Therefore, the caveolae deform and the density of caveolin-1 changes, but the change is continuous instead of stepwise. Thus, the dispersed caveolin-1 (in the first density range) is thought to be outside the caveolae because of the mutually exclusive localizations.

### The density distribution of caveolin-1 upon methyl-β-cyclodextrin and dynasore treatment

**Figure 5:**
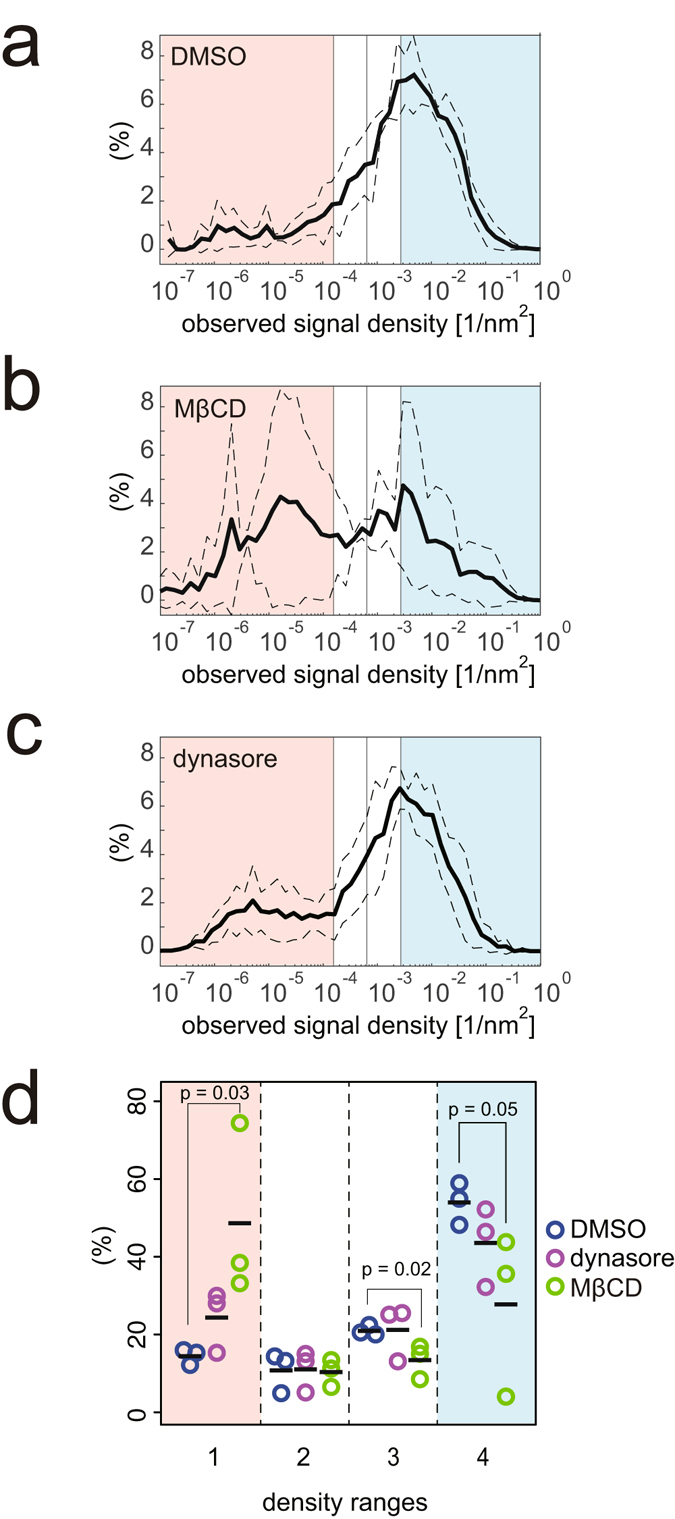
Local density estimation from STORM signals of caveolin-1 upon MβCD or dynasore treatment. (**a**–**c**) The average density distributions of the STORM signals by the 4^th^ NND of caveolin-1 under (**a**) control DMSO treatment, (**b**) 10-mM MβCD treatment for 10 min, and (**c**) 80-μM dynasore treatment in isotonic medium for 10 min, as analysed in Fig. 3. (**d**) Percentages of caveolin-1 by the density ranges in each cell as in Fig. 3f. The average percentages at the dispersed caveolin-1 range between cells treated with DMSO and MβCD were significantly different. Indicated p-values were obtained using the Student’s t-test.

Caveolae are also considered to be cholesterol-dependent structures, such that the depletion of cholesterol is known to flatten caveolar structures^6, 7^. However, the dispersion of caveolin-1 upon cholesterol-depletion has not been examined by super-resolution microscopy. In cells treated with methyl-β-cyclodextrin (MβCD), the percentage at the dispersed caveolin-1 density (in the first range) significantly increased (Figs 5a,b,d and S2). However, the percentage at the flattened caveolar density (in the second range) was not increased.

**Figure 6:**
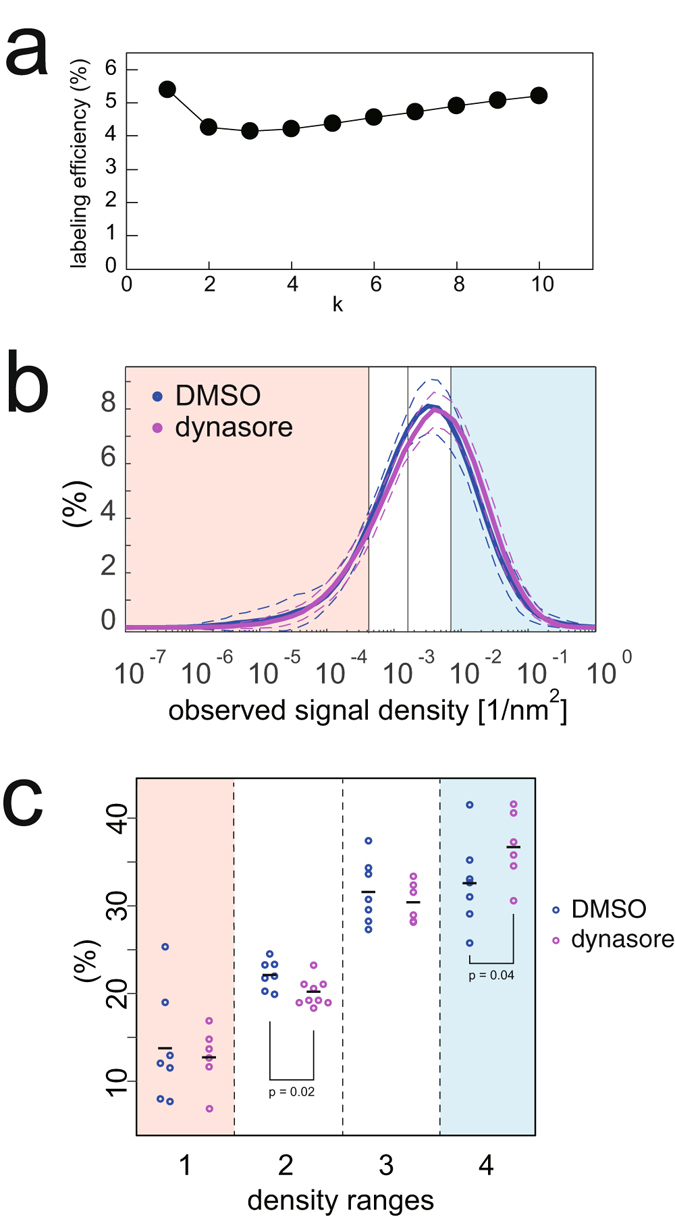
Local density estimation from STORM signals of clathrin heavy chain. (**a**) Observation efficiency estimations of clathrin heavy chain with the *k* ^th^ NND plotted against k. (**b**) The average density distributions of the STORM signals by the 8^th^ NND of clathrin heavy chain in cells under isotonic culture medium (blue), and 80 μM dynasore treatment for 10 min (magenta), as analysed in Fig. 3. The dashed line indicates the standard deviation. (**c**) Percentages of clathrin heavy chain by the density ranges in each cell allocated as in Fig. 3f. The average percentages at the flattened range (second range) and the high-density range (fourth range) between cells treated with DMSO and dynasore were significantly different. Indicated p-values were obtained using the Student’s t-test.

To further verify the method described here for the detection of protein densities of small structures on cell plasma membranes, we labelled the clathrin heavy chain with an antibody and evaluated its protein density. Clathrin-coated pits have a diameter of approximately 150 nm and contain approximately 100 clathrin triskelions, i.e., 300 clathrin heavy chain proteins^19^. Approximately 4% of clathrin heavy chain proteins were labelled in a selected cell (Fig. 6a). We then labelled the clathrin heavy chain proteins in dynasore-treated cells. The high-density population of the clathrin heavy chain proteins (in the fourth range) increased upon dynasore treatment (Figs 6b,c and S3), which suggested that our method was valid for the measurement of protein assembly on the plasma membrane. By contrast, we could not detect any change in the density distribution of caveolin-1 after dynasore treatment (Figs 5a,c,d and S2).

### The density distribution of caveolin-1-Dendra2

**Figure 7:**
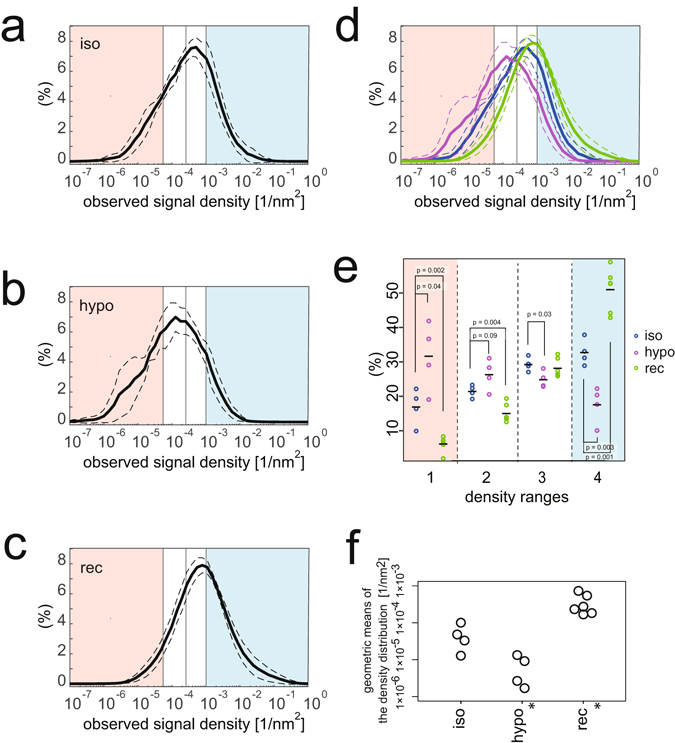
Local density estimation from PALM signals of caveolin-1-Dendra2. (**a**–**d**) The average density distributions of the PALM signals by the 4^th^ NND of caveolin-1-Dendra2-expressing cells under (**a**) isotonic medium (iso), (**b**) hypotonic treatments of 5 min (hypo), and (**c**) hypotonic treatments of 5 min followed by isotonic medium for 5 min recovery (rec), as analysed in Fig. 3. The dashed line indicates the standard deviation. The dotted line indicates the peak in (**a**). In (**d**), the superposition of (**a**–**c**) is shown. (**e**) Percentages of caveolin-1 by the density ranges in each cell as in Fig. 3f. The average percentages at the dispersed caveolin-1 range between cells under isotonic medium and cells treated with hypotonic medium or recovered cells were significantly different. Indicated p-values were obtained using the Student’s t-test. (**f**) The geometric means of the density distribution in each cell in (**a–c**). The average geometric means between cells in isotonic medium and the treated cells were significantly different (*) with p = 0.02 (hypo) and 0.002 (rec) obtained using the Student’s t-test.

Antibody labelling detects endogenous protein but requires solubilization of the plasma membrane for incorporation of the antibody into the cells, a procedure called ‘permeabilization’. By contrast, proteins with a genetically coded tag, such as Dendra2, do not need permeabilization of the plasma membrane for their visualization. The other conditions for the observations were similar between the antibody labelling method and the fluorescent protein observation. We observed HeLa cells expressing low levels of caveolin-1-Dendra2, a photoswitchable protein, as previously performed for caveolin-1-mCherry^20^. Because the expression of caveolin-1-Dendra2 varied cell-to-cell, we averaged the density distribution of caveolin-1 under the same treatment using the mode as the standard, as in Fig. 3 (Figs 7 and S4). As shown in Fig. 7a, the density distribution of caveolin-1-Dendra2 under the isotonic condition was similar to the density distribution of caveolin-1 determined by antibody labelling in Fig. 3; however, the averaged mode density was less than that observed by antibody labelling, presumably due to exogenous expression and the lesser efficiency of photon emission of photoswitchable proteins compared with fluorescent antibodies^21, 22^.

The peak density of caveolin-1-Dendra2 decreased greatly in cells under hypo-osmotic treatment, with an increase of dispersed caveolin-1 (in the first range) (Fig. 7b,d,e). Moreover, the one-quarter-density caveolin-1 (in the second range) was increased in the observations using Dendra2 (Fig. 7b,d,e). High-density caveolin-1-Dendra2 structures were detected (Fig. 7b), but their population was decreased. Because caveolin-1-Dendra2 was visualized without solubilization of the plasma membrane (permeabilization), the non-increase in the one-quarter-density caveolin-1 by antibody staining (Fig. 3f) presumably resulted from the solubilization and removal of caveolin-1 from the cells during the antibody staining procedure.

Disassembled caveolae have been reported to recover after cells were replenished with an isotonic medium^4^. After recovery from the hypo-osmotic treatment, the distribution of caveolin-1-Dendra2 was recovered to the high density (Fig. 7c,f). We next calculated the geometric means of the density distribution. The geometric means of the hypo-osmotic treated cells and the recovered cells were significantly different from the geometric mean of the cells in the isotonic medium (Fig. 7f). Thus, these data suggested that our measurement of caveolin-1 density by caveolin-1-Dendra2 clearly detected the deformation and the recovery of caveolae.

## Discussion

Membrane tension induced by hypotonic conditions mechanically disassembles caveolae, which buffer membrane tension by their disassembly^4, 23^. The detailed behaviour of caveola deformation has not been statistically described. In this study, we used the coordinates of caveolin-1, obtained with STORM and PALM, to determine the two-dimensional projection density of caveolin-1 in both hypo-osmotic treated cells and cells under normal isotonic culture conditions; as such, we quantitatively measured the deformation of caveolae. Using a nearest-neighbour distance (NND)-based density measurement, we detected the disassembly of caveolin-1. Surprisingly, the antibody labelling method revealed that the percentage of one-quarter-density caveolin-1, i.e., caveolin-1 at the flattened caveolar density, was not increased after hypo-osmotic treatment. In contrast, the percentage of one-quarter-density caveolin-1 was increased when we observed caveolin-1 fused with Dendra2 without antibody labelling. The difference in procedures included the presence or absence of solubilization of the plasma membrane. Therefore, upon hypo-osmotic treatment, caveolin-1 is suggested to rapidly disassemble, which increases its propensity for detergent solubilization.

The multivariate Ripley’s L function analysis indicated that only the dispersed caveolin-1 (first range in Figs 3 and 7) forms a distinct population from the caveolin-1 in the typical, high-density, and flattened caveolae (second, third, and fourth ranges in Figs 3 and 7). However, the increase in caveolin-1 in the first and second density ranges in Fig. 7 suggested that the caveolin-1 in the caveolae dispersed into the sparsely distributed caveolin-1 after hypo-osmotic treatment possibly through flattened caveolar intermediates.

We compared our observations with those from previous studies. In previous studies, the decrease of caveolin-1 from the caveolar invagination and the increase of caveolin-1 on the flat membrane area have been demonstrated in mouse lung endothelial cells (MLEC) by thin-section electron microscopy, which could not distinguish the flattened caveolae from the flat, non-caveolar plasma membrane^4^. The increase in flattened caveolae in MLEC was consistent with detailed observations of the plasma membrane using the rapid-freeze deep-etch electron microscopy, in which caveolae were assessed based on their characteristic surface striations^4^. However, sparsely distributed plasma membrane-associated caveolin-1 could not be visualized by rapid-freeze deep-etch electron microscopy. In contrast, the freeze-fracture immuno-electron microscopy method can observe membrane-embedded molecules including proteins and lipids, that were immobilized by platinum-coating from the inner hydrophobic faces of the bilayer membrane^24, 25^. With this method, the caveolin-1 outside of the invaginated membrane structures, which appeared to be equivalent to dispersed caveolin-1 in our observations, was observed in human fibroblast cells^17^. However, this freeze fracture method had not been applied for the hypo-osmotic treated cells. Decreased caveolin-1 in the plasma membrane after hypo-osmotic treatment was previously observed in HeLa cells using conventional microscopy^4^; however, dispersed caveolin-1 would be concealed by background fluorescence. In all these studies, there was no clear characteristics to distinguish flattened and typical caveolae. Therefore, our observations by density estimation do not contradict previous reports.

The high-density caveolin-1 existed after hypo-osmotic treatment, and therefore, some caveolin-1 population remained in high density after membrane tension. The high-density caveolin-1 revealed by the projection density measurement may represent the assembly of caveolin-1 perpendicular to the plasma membrane. Thus, the high-density caveolin-1 may be achieved by the constriction of caveolae. The constriction may occur during endocytosis. Caveolae and clathrin-mediated endocytosis are known to be mediated by the GTPase dynamin^7^. However, the treatment of the cells with the dynamin-inhibitor, dynasore, did not increase the amount of high-density caveolin-1 (Fig. 5). Because we detected an increase in high-density clathrin following dynasore treatment (Fig. 6), it is possible that the small deformation in caveolae caused by dynasore treatment would be undetectable due to the smaller size of caveolae compared to clathrin-coated pits. The absence of an increase in high-density caveolin-1 following dynasore treatment could also result from less-frequent endocytosis of caveolae than clathrin-coated pits^26^ or from the difference in the steps before scission by dynamin. Clathrin-coated pits are thought to undergo several maturation steps before dynamin-mediated scission^19^. The increase in the high-density population of clathrin upon dynamin inhibition is consistent with multiple steps of clathrin-coated pit maturation. By contrast, caveolae do not appear to have such multiple steps of maturation before dynamin-mediated scission^7^. Thus, dynamin inhibition is not thought to increase the high-density caveolin-1 population. Alternatively, the high-density caveolin-1 may have resulted from the dynamin-independent physical behaviour of caveolae under tension. This concept was previously discussed by Sens and Turner using a theoretical model that may explain the condensed caveolin-1 and the increase in free caveolin-1^27^.

The responses of caveolae to hypo-osmotic tension may also be specific to cell type, i.e., caveolae have cell-type-specific characteristics. Cavin proteins are important structural components of caveolae as the removal of Cavin proteins leads to the disassembly of caveolae^7^. Cavin proteins have several family members and different functions that are dependent on cell type. Cavin proteins from different tissues and cells have been differentially fractionated by density gradient centrifugation analysis^28, 29^. Caveolin isoforms are also suggested to be differentially localized dependent on the deepness of caveolae^17^. Thus, the responses of caveolae after the application of tension by hypo-osmotic treatment included flattening of the caveolae and the dispersion of caveolin-1 throughout the plasma membrane, and these responses are thought to depend on the cell-specific protein composition of the caveolae. The hypo-osmotic tension activates PKC, which induces the removal of PACSIN2, an F-BAR protein known to stabilize caveolae and bind to dynamin, by PKC-mediated phosphorylation under both hypotonic and isotonic conditions^20, 30^. The molecular details of caveola disassembly upon hypo-osmotic treatment will be further examined. Dysfunctions in caveolae have been found in various diseases, such as muscular dystrophy^7^. Our detailed analysis of caveolin-1 distribution after membrane tension provides fundamental insights for understanding the behaviour of caveolae.

## Materials and Methods

### Immunofluorescence for STORM

HeLa cells were cultured as described previously^31^. For the hypotonic treatment, Dulbecco’s modified Eagle’s medium (DMEM), supplemented with 10% foetal calf serum (FCS), was diluted with sterile water in a 1:10 ratio^4^. The three-dimensional STORM setup was purchased from Nikon, and a protocol was developed based on previous reports^11, 12^. Dye preparation, secondary antibody labelling and cell staining for STORM imaging (Nikon) were performed according to the manufacturer’s protocol, using combinations of Alexa 405 and Alexa 647^11, 12^. Cells were cultured on Lab-Tek II chambered cover glass slides that were precleaned with 1 M KOH for 1 hr. The cultured cells were fixed in 3% paraformaldehyde and 0.1% glutaraldehyde (electron microscopy grade) in HEPES-buffered saline for 10 min, reduced with 0.1% NaBH_4_ in HEPES-buffered saline for 7 min, blocked in blocking buffer (3% BSA + 0.2% Triton X-100 in PBS) for 1 hr, and then stained with a 1:100 dilution of the primary antibody, anti-caveolin-1 (7C8) mouse monoclonal antibody (sc-53564, Santa Cruz Biotechnology) or anti-clathrin heavy chain (TD.1) mouse monoclonal antibody (sc-12734, Santa Cruz Biotechnology), in blocking buffer for 1 hr. After being washed, the cells were incubated with the secondary antibody and then washed again. Finally, the cells were post-fixed with 4% paraformaldehyde + 1% glutaraldehyde and stored in PBS at 4 °C. For image acquisition, the cells were soaked in 50 mM Tris-HCl (pH 8.0), 10 mM NaCl, and 10% glucose supplemented with cysteamine (MEA), glucose oxidase and catalase for caveolin-1 or, for clathrin staining, in the modified buffer described previously^32^, and imaged using an N-STORM setup with a Piezo stage and an iXon DU-897E electron-multiplying charge-coupled device (EMCCD) camera (Andor) according to the manufacturer’s instructions. One image for the activation laser (405 nm) and three sequential images for the reporter laser (647 nm) were obtained for 10,000 cycles (a total of 40,000 images). Only the signals identified by the activation laser within 25 nm in both images and the first images by the reporter laser were considered for the analysis. The cylindrical lens was inserted into the light path to obtain the z-axis coordinates and was used to reject ambiguous signals. The signals were analysed with the manufacturer’s software (NIS-elements, Nikon). The coordinates of the signals identified by the software were exported into a text file and then analysed. In all figures, each dot represented one molecule. The illumination depth did not exceed 500 nm from the coverslip and was considered on or within close vicinity of the plasma membrane.

### The multivariate Ripley’s L function

STORM signals were classified into four ranges based on the signal density surrounding the focal signals (kth nearest neighbour distances for the focal signals are used to estimate the signal density). Let ${{\boldsymbol{x}}}_{n}^{i}$ be the position of the *n*th signals in *i*th ranges. We first defined the multivariate K function as

3 $${K}_{ij}(d)=\frac{1}{{N}_{i}}\sum _{n}^{{N}_{i}}\sum _{m}^{{N}_{j}}H(d-|{{\boldsymbol{x}}}_{n}^{i}-{{\boldsymbol{x}}}_{m}^{j}|)$$

where *d* is the length (in nanometres), *N*_*i*_ is the number of signals in the *i*th range, and *H* is the Heaviside function. Then, we calculated the multivariate Ripley’s L function as

4 $${L}_{ij}(d)=\sqrt{\frac{{K}_{ij}(d)}{{K}_{ij}(5000)}}-\frac{d}{5000}.$$

This function is normalized to take *L*_*ij*_(5000) = 0 by assuming the correlations of spatial signal patterns disappear at the length scale of 5000 nm.

### PALM imaging of Dendra2

Flp-in T-REx HeLa cells were cultured in DMEM supplemented with 10% FCS, and caveolin-1-Dendra2 was expressed using the Flp-In T-REx Core Kit (Thermo Fisher Scientific) as described previously^20, 33, 34^. Cells fixed as above without permeabilization were reduced with PBS +0.1% NaBH_4_ for 7 min, washed, and then stored in PBS with 1% polyvinyl alcohol and 10 mM cysteamine (MEA). Cells with weak expression of caveolin-1 were selected based on fluorescence intensity. Then, the cells were imaged using the N-STORM setup. Data recording was performed using NIS-elements software. Forty thousand images were continuously acquired using a 30 mW 543 nm laser with continuous activation by a 405 nm laser. The coordinates of the signals, identified by the software, were exported into a text file and then analysed.

## Electronic supplementary material

Supplementary Figures and Legends
