# Supplementary material for: Measurement of caveolin-1 densities in the cell membrane for quantification of caveolar deformation after exposure to hypotonic membrane tension
Source: Sci Rep. 2017 Aug 10;7:7794. doi: 10.1038/s41598-017-08259-5 (PMC5552771; doi:10.1038/s41598-017-08259-5)
Supplement: Supplementary file 1 — Supplementary Figures and Legends [file 41598_2017_8259_MOESM1_ESM.pdf]

Supplementary Information for

Measurement of caveolin-1 densities in the cell membrane for quantification of caveolar deformation after exposure to hypotonic membrane tension

Masashi Tachikawa[1*], Nobuhiro Morone[2], Yosuke Senju[4], Tadao Sugiura[3], Kyoko Hanawa-Suetsugu[5], Atsushi Mochizuki[1], Shiro Suetsugu[5*]

[1] Theoretical Biology Laboratory, RIKEN, 2-1 Hirosawa, Wako 351-0198, Japan

[2] MRC Toxicology Unit, University of Leicester, Leicester LE1 9HN, UK, and Institute for Integrated Cell-Material Sciences (iCeMS), Kyoto University, Kyoto 606-8501, Japan

[3] Graduate School of Information Science, Nara Institute of Science and Technology, Ikoma 630-0192, Japan

[4] Institute of Biotechnology, University of Helsinki, Helsinki 00014, Finland

[5] Graduate School of Biological Sciences, Nara Institute of Science and Technology, Ikoma 630-0192, Japan

Supplementary Figure Legends

Figure S1: The caveolin-1 density distributions in cells under isotonic and hypotonic conditions (related to Figure 3).

The heat maps of the caveolin-1 density, the density distributions by the kth NND measurement at $k = 2$, 4, and 6 and the multivariate Ripley's L functions are shown. The heat maps were generated from the original signal position data, with each point replaced by a Gaussian kernel with $\sigma = 400[\mathrm{nm}]$. The specificity of the signals was examined by either omitting the primary anti-caveolin-1 antibody or knocking downcaveolin-1 through RNAi.

Figure S2: The caveolin-1 density distributions in cells upon MβCD or dynasore treatment (related to Figure 5).

The heat maps of the caveolin-1 density, the density distributions by the kth NND measurement at $k = 2$, 4, and 6 and the multivariate Ripley's L functions are shown as Figure S1.

Figure S3: The clathrin-heavy chain density distributions in cells under isotonic and hypotonic conditions (related to Figure 6).

The heat maps of the clathrin-heavy chain, the density distributions by the kth NND measurement at $k = 2$, 4, 6, and 8 and the multivariate Ripley's L functions are shown are shown as Figure S1.

Figure S4: The caveolin-1-Dendra2 density distributions in cells under isotonic and hypotonic conditions (related to Figure 7).

The heat maps of the caveolin-1-Dendra2 density, the density distributions by the kth NND measurement at $k = 2$, 4, and 6 and the multivariate Ripley's L functions are shown as Figure S1.

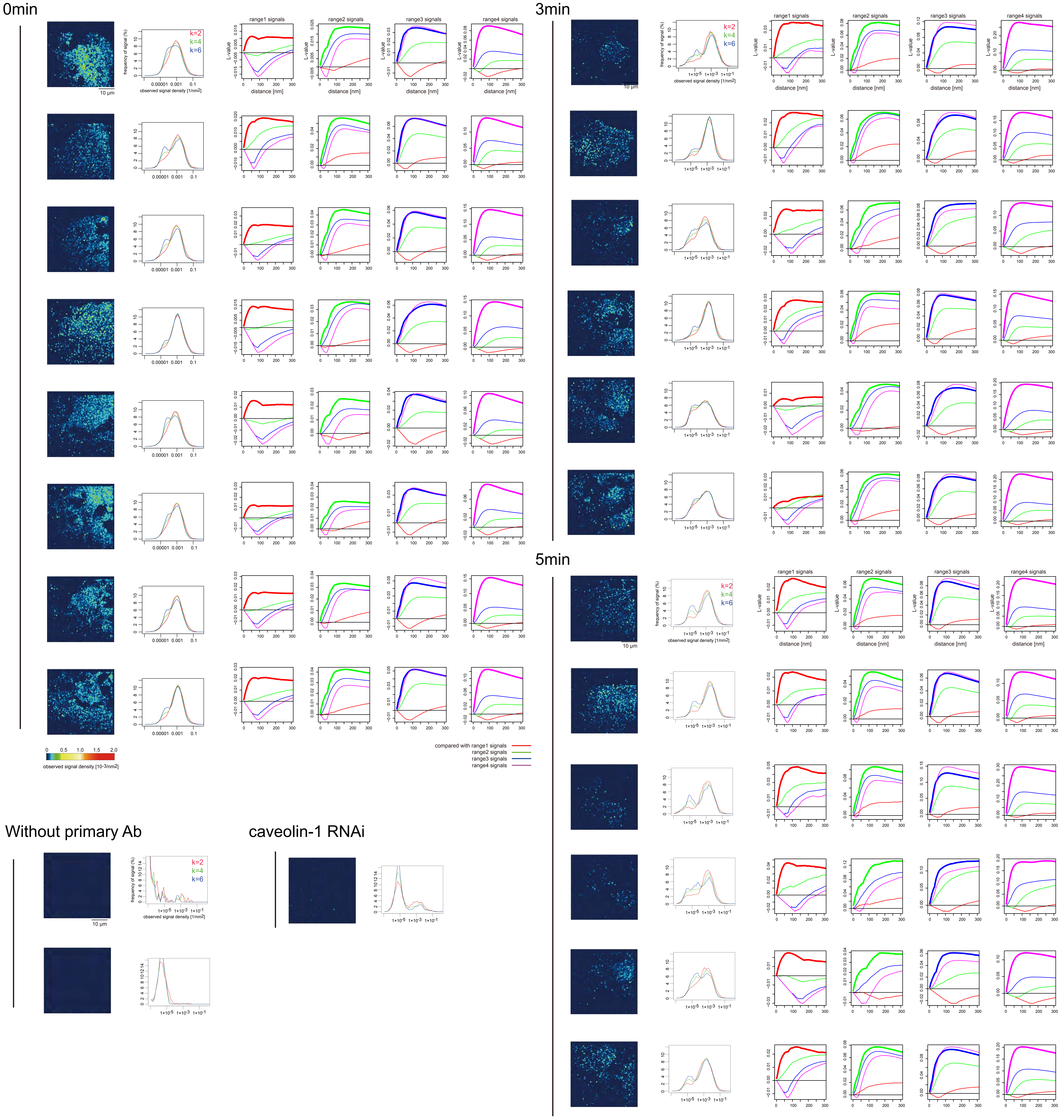
0min
3min
5min
Without primary Ab
caveolin-1 RNAi
range1 signals
range2 signals
range3 signals
range4 signals
k=2
k=4
k=6
frequency of signal (%)
observed signal density [1/nm2]
L-value
distance [nm]
10 µm
0
0.5
1.0
1.5
2.0
observed signal density [10-3/nm2]
compared with range1 signals
range2 signals
range3 signals
range4 signals

Figure S1

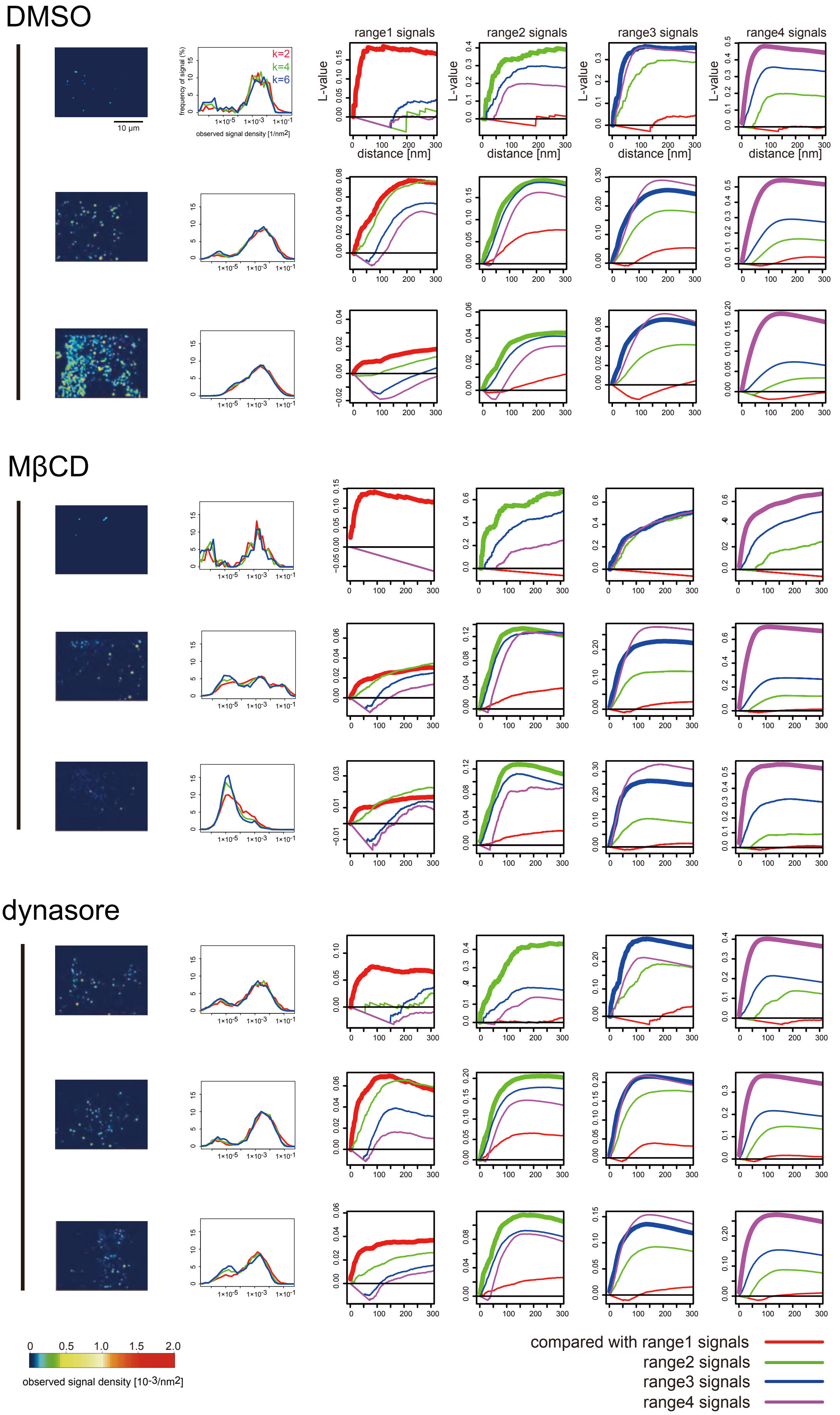
DMSO
MβCD
dynasore
range1 signals
range2 signals
range3 signals
range4 signals
L-value
distance [nm]
frequency of signal (%)
observed signal density [1/nm2]
k=2
k=4
k=6
10 μm
observed signal density [10-3/nm2]
compared with range1 signals
range2 signals
range3 signals
range4 signals

Figure S3

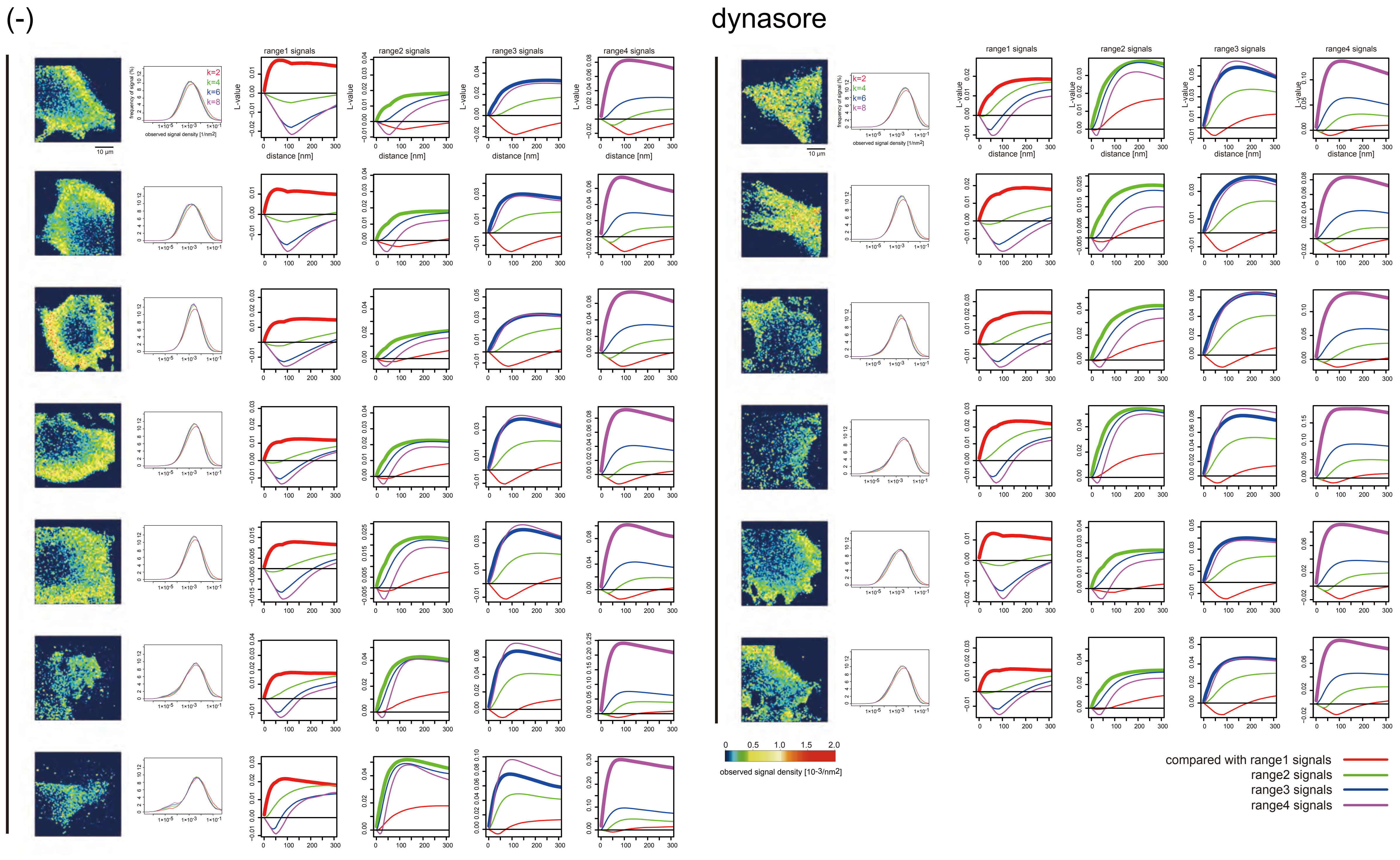
(-)
dynasore
range1 signals
range2 signals
range3 signals
range4 signals
L-value
distance [nm]
frequency of signal (%)
observed signal density [1/nm2]
k=2
k=4
k=6
k=8
10 μm
0 0.5 1.0 1.5 2.0
observed signal density [10-3/nm2]
compared with range1 signals
range2 signals
range3 signals
range4 signals

Figure S4

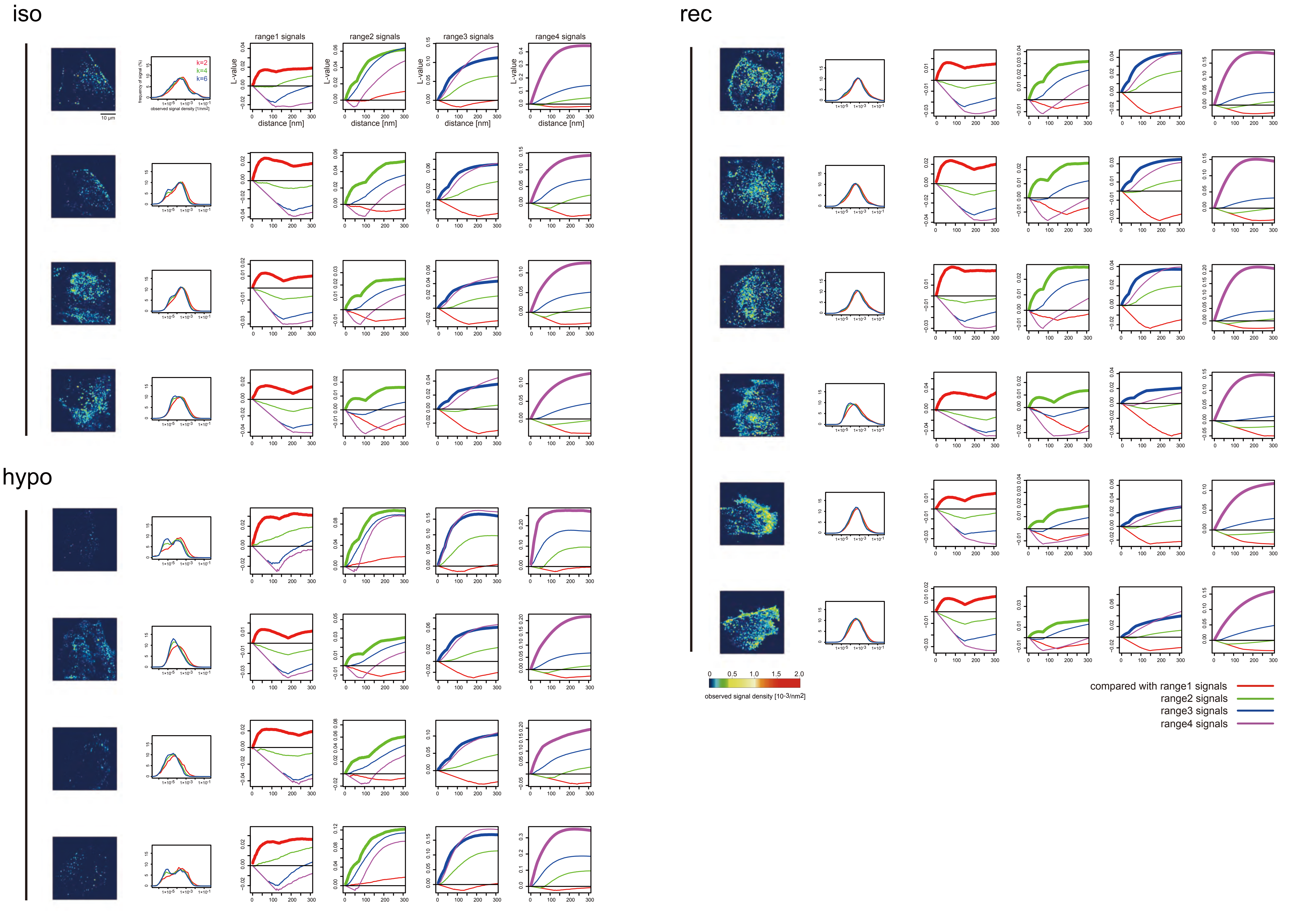

iso
hypo
rec
range1 signals
range2 signals
range3 signals
range4 signals
L-value
distance [nm]
k=2
k=4
k=6
10 µm
observed signal density [10-3/nm2]
compared with range1 signals
range2 signals
range3 signals
range4 signals

Figure S5
